# DNA Sequence Changes Resulting from Codon Optimization Affect Gene Expression in *Pichia pastoris* by Altering Chromatin Accessibility

**DOI:** 10.3390/jof11040282

**Published:** 2025-04-03

**Authors:** Chaoyu Lu, Linna Guo, Bohao Fang, Jiacheng Shi, Mian Zhou

**Affiliations:** State Key Laboratory of Bioreactor Engineering, East China University of Science and Technology, Shanghai 200237, China; y30220487@mail.ecust.edu.cn (C.L.); y30230452@mail.ecust.edu.cn (B.F.); y30200490@mail.ecust.edu.cn (J.S.)

**Keywords:** codon usage bias, nucleosome occupancy, chromatin accessibility, *Pichia pastoris*

## Abstract

Codon optimization is a widely employed strategy to enhance protein expression. However, it occasionally leads to unexpected transcriptional repression despite preserving amino acid sequences. This study investigates the mechanistic basis of such transcriptional attenuation by analyzing two gene candidates (*0432* and *Fluc*) in the common expression chassis *P. pastoris*. Both genes experienced severe mRNA reduction following codon optimization. Evidenced by histone H3 chromatin immunoprecipitation (ChIP) and a DNase I hypersensitivity assay, gene sequences with transcriptional repression displayed elevated nucleosome occupancy and reduced chromatin accessibility. The above change was caused by an ORF sequence change independent of the promoter, since transcriptional attenuation and compromised chromatin accessibility were still observed after replacing the strong promoter P*_GAP_* with P*_por1_* or P*_rps8b_*. Our findings challenge the conventional view of codon optimization as solely translation-centric, revealing its capacity to preemptively modulate transcription through chromatin accessibility. This work underscores the necessity of integrating chromatin-level considerations into synthetic gene design to avoid unintended transcriptional silencing and optimize expression outcomes.

## 1. Introduction

Codon usage bias refers to the phenomenon where certain synonymous codons are preferentially used during amino acid encoding, and this occurs in all known organisms [1,2,3]. Studies have shown that codon usage bias plays an important role in regulating gene expression levels [4,5,6,7]. For example, during mRNA translation, codon usage bias affects translation initiation and elongation rates [8,9], thereby influencing protein expression levels. Additionally, it impacts co-translational protein folding by modulating the ribosomal elongation rate, which ultimately affects the structure and function of the protein [8,9,10].

Optimizing the codons of a foreign gene to match the preferred codons of the host cell has traditionally been an effective method to enhance heterologous protein expression [11,12]. For example, in the commonly used yeast expression system *Pichia pastoris*, applying a codon optimization strategy to express the α-amylase gene fragment from the bacterium *Geobaccilus* sp. led to a maximum 40% increase in expression [13]. Similar enhancements were observed when codon-optimized 1,3-1,4-beta-D-glucanase showed 38.5% higher yields in *Pichia pastoris* expression systems [14], and codon-optimized porcine β-defensin–2 (pBD2) achieved 4–6 times increased secretion in *E. coli* [15]. However, some other genes showed an unexpected drop in expression levels after codon optimization [16,17,18], such as codon-optimized *PAS_FragD_0013*, *PAS_chr1*–*1_0135*, and *PAS_chr1*–*4_0616* [19]. More issues arise at the transcriptional level rather than the translational level.

Although not altering the amino acid sequence, codon optimization changes the gene DNA sequence. Therefore, it was possible to affect the mRNA secondary structure and stability [20]. In addition, a few studies have focused on the preliminary role of codon optimization on transcription. In *Neurospora crassa*, optimal codon usage was negatively correlated with the epigenetic silencing marker H3K9me3 [7,21], as well as pre-mature transcription termination events [22]. In *E. coli*, codon optimization regulated transcription by altering cytosine methylation sites [23]. However, additional mechanisms are still waiting to be revealed.

Gene transcription activity is closely correlated with the chromatin structure, referred to as chromatin accessibility [24]. For example, transcription is typically active during interphase, when chromatin is more open to allow RNA polymerase binding and elongation [25]. Chromatin accessibility is usually determined by the DNA packaging pattern, which is then tightly related to the arrangement and density of nucleosomes [26,27,28]. Classic studies demonstrated that nucleosome positioning directly modulates transcription factor binding and Pol II processivity [29]. Nucleosome-dense regions form compact heterochromatin that sterically hinders transcription machinery, while accessible euchromatin permits RNA polymerase II (Pol II) elongation [30,31]. DNA sequences, histone modification, as well as non-coding RNA interference are possible factors affecting nucleosome stability [32,33,34,35,36]. However, the relationship between the synonymous codon choice, nucleosome pattern, and chromatin accessibility remains obscure.

In our previous study, codon optimization was performed on a series of endogenous and heterologous genes in *P. pastoris* to study its role on transcription [16,19,37]. Among them, two candidates (*PAS_chr2-2_0432* [16] and *firefly luciferase* [37]) attracted further attention, since codon optimization severely decreased their mRNA levels. Here, we then carefully examined the cause of an mRNA drop after codon optimization. Histone density and chromatin accessibility were measured under different promoter strengths to unveil any possible principles.

## 2. Materials and Methods

### 2.1. Strains and Culture Conditions

*E. coli* DH5α strains were used for plasmid construction and propagation. *E. coli* cells were cultured in LB containing 0.5% yeast extract, 1% tryptone, and 1% NaCl at 37 °C with 100 µg/mL of ampicillin when required.

*P. pastoris* strain *GS115* was used as the wild type and the host for transgenic strain generation. All cultured cells were shaken at 220 rpm and 30 °C. For seed preparation, yeast cells were inoculated into 2 mL YPD medium (2% tryptone, 1% yeast extract, 2% glucose) until OD_600_ reached 2–8. For the shake flask culture, cells were then inoculated into 50 mL YPD with initial OD_600_ = 1 and collected until OD_600_ = 12–15.

### 2.2. Plasmid Construction and Strain Generation

The *0432-ori* sequence was PCR-amplified from the *P. pastoris* genome. *0432-opt*, *FLUC-ori*, and *FLUC-opt* sequences were directly synthesized by GENEWIZ. For *0432-optTGA*, a TGA stop codon was inserted right after the 271st codon of *0432-opt*. All ORFs were cloned into the pGAPZ vector, keeping the C-terminal 6 × His tag. To replace P*_GAP_* with P*_por1_* and P*_r__ps8b_*, promoter sequences were again PCR-amplified from the *P. pastoris* genome and ligated with the rest of the construct. The resulting plasmids were linearized at the *his4* locus and electroporated into the *GS115* wild-type strain. Positive transformants with a single integration copy were verified by PCR. All ORFs and primer sequences are listed in the Supplementary Materials.

### 2.3. RNA Extraction and RT-qPCR

Yeast cells were collected by centrifugation and washed by PBS twice. Cells were then lysed in TRIeasy™ Total RNA extraction reagent (Yeasen, Shanghai, China, #10606) with a mechanical homogenizer (YeTuo, Shanghai, China), and centrifuged at 12,000× *g* for 10 min. RNA was extracted via phenol–chloroform purification, precipitated with ethanol at −80 °C, and resuspended in DEPC-treated water. For each sample, 1 μg total RNA was used for cDNA synthesis (Hifair^®^ III 1st Strand cDNA Synthesis SuperMix for qPCR, Yeasen, #11141) and subsequent quantitative PCR (Hieff UNICON^®^ Universal Blue qPCR SYBR Green Master Mix, Yeasen, #11184). The qPCR program was set as follows: step 1: 95 °C for 2 min; step 2: 95 °C for 10 s; step 3: 60 °C for 30 s; and repeat steps 2–3 for 39 cycles. Primers for amplification were designed with the help of SnapGene 6.0.2.

### 2.4. Chromatin Immuno-Precipitation (ChIP) Assay and qPCR

Yeast cells were cultured in YPD medium at 30 °C for the stationary phase (OD_600_ = 12–15). Cells were crosslinked with 1% formaldehyde for 10 min at 30 °C, quenched with 0.1 M glycine (final concentration), and washed twice with ice-cold PBS. Cell pellets were flash-frozen in liquid nitrogen and ground into fine powder. Chromatin was solubilized in ChIP buffer (50 mM HEPES pH 7.4, 150 mM NaCl, 1 mM EDTA, 0.25% Triton X-100, 0.5% NP-40) and sonicated to generate DNA fragments of 200–800 bp, verified by 2% agarose gel electrophoresis. After centrifugation (12,000 rpm, 15 min, 4 °C), supernatants were adjusted to a 2 mg/mL protein concentration (Bradford assay) for immunoprecipitation.

Pre-clearing was performed by incubating 1 mL chromatin lysate with 20 μL pre-blocked Protein G beads (GenScript, Nanjing, China, #L00209) which were pre-washed with ChIP buffer and blocked with 1% BSA for 1–2 h at 4 °C. For immunoprecipitation, pre-cleared lysates were incubated overnight at 4 °C with 2–4 μg The histone H3 antibody (Histone H3 Rabbit mAb, Abclonal, Wuhan, China, #A17562) or H3K27me3 antibody (TriMethyl-Histone H3-K27 Rabbit mAb, Abclonal, #A22006) per 25 μg chromatin was used. Immune complexes were captured with 30 μL blocked Protein G beads (1–2 h, 4 °C), followed by sequential washes with low-salt buffer, high-salt buffer, LNDET buffer, and TE buffer. Bound chromatin was eluted in ChIP Elution Buffer, and crosslinks were reversed by 65 °C incubation with 0.2 M NaCl for 12–16 h (detailed formulations are provided in reference [38]).

Input samples (10% of lysate) and immunoprecipitated DNA were treated with RNase A (37 °C, 1 h), purified by phenol–chloroform extraction, and precipitated with ethanol. DNA pellets were resuspended in 20 μL TE buffer and analyzed by qPCR using gene-specific primers; see qPCR operation in Section 2.3. Data were normalized to input controls and quantified as fold enrichment over background.

### 2.5. Chromatin Accessibility Assay

Yeast cells were collected by centrifugation, washed twice with PBS, and flash-frozen in liquid nitrogen. Cell pellets were ground and resuspended in Buffer A (1 M sorbitol, 7% ficoil, 20% glycerol, 5 mM MgAc, 5 mM EGTA, 3 mM CaCl_2_, 3 mM DTT, 50 mM Tris-HCl, pH 7.5), followed by homogenization in Buffer B (25% glycerol, 5 mM MgAc, 5 mM EGTA, 25 mM Tris-HCl, pH 7.5). Chromatin was purified by sucrose gradient centrifugation (1 M sucrose, 10% glycerol, 5 mM MgAc, 1 mM DTT, 25 mM Tris-HCl, pH 7.5) at 15,000× *g* for 15 min at 4 °C.

Purified chromatin was resuspended in DNase Buffer containing protease inhibitors and divided into two aliquots for DNase I (Yeasen, #10325) treatment or the untreated control. Reactions were terminated with stop buffer (Yeasen, #10325) and incubated at 65 °C for 10 min. Samples were treated with 0.5% SDS and at 70 °C for 15 min and digested with RNase A (TIANGEN, Beijing, China, #RT405) and Proteinase K (TIANGEN, #RT403). DNA was purified by phenol–chloroform extraction, precipitated with ethanol, and resuspended in 50 μL TE buffer. qPCR was performed using primers targeting specific gene regions. Ct values were normalized to untreated controls, and chromatin accessibility was calculated as the relative loss of amplification efficiency in DNase I-treated samples [39].

### 2.6. Data Analysis and Statistics

In the qPCR for measuring the transcriptional level, *GAPDH* (*PAS_chr2-1_0437*) was selected as the internal control. mRNA levels were normalized with primer efficiency. Statistical analysis and graph plotting were performed using GraphPad Prism 9 software. Error bars represent the standard error of the mean (SEM). Statistical significance was determined by t-tests, with significance levels divided according to *p*-values of >0.05 (ns), 0.05–0.01 (*), 0.01–0.001 (**), 0.001–0.0001 (***), and <0.0001 (****).

## 3. Results

### 3.1. Codon Optimization Severely Abolished Mature mRNAs of 0432 and Fluc

**Figure 1:**
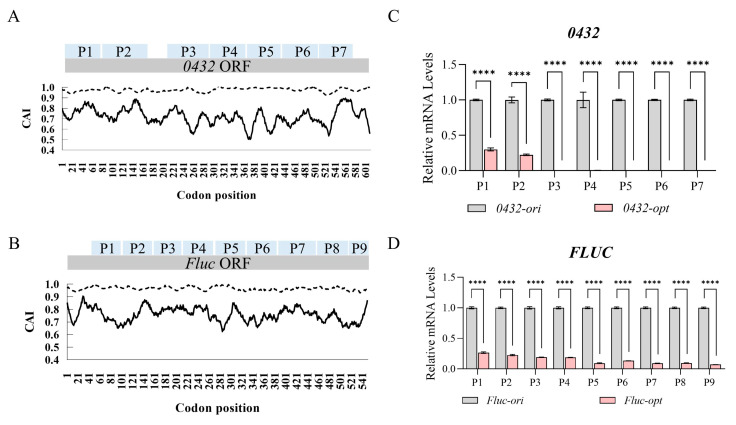
Codon optimization severely abolished mature mRNAs of *0432* and *Fluc*. (**A**,**B**) Codon Adaptation Index (CAI) curves of *0432* (**A**) and *Fluc* (**B**) before (solid line) and after (dashed line) codon optimization. P1–P7 in the *0432* ORF (**A**) and P1–P9 in the *Fluc* ORF (**B**) denote specific detection regions, which are used to characterize the positional distribution of each tested area within the open reading frame. (**C**,**D**) RT-qPCR comparing mRNA levels of *0432* (**C**) and *Fluc* (**D**) after codon optimization; N = 3. For each gene, a series of primer sets along the ORF was tested (P1, P2…). For each primer set, the RNA level of the original sequence was set to 1. *p*-values: <0.0001 (****).

Among all the genes we have tested so far, the endogenous gene *PAS_chr2-2_0432* (hereafter *0432*, coding for a regulatory subunit of the *Atg1p* signaling complex) and heterologous gene *firefly luciferase* (hereafter *Fluc*) exhibited the most prominent transcription defects following codon optimization. Both genes were optimized based on the codon usage bias of *Pichia pastoris*, with the Codon Adaptation Index (CAI, a metric reflecting the similarity of codon usage frequencies to highly expressed host genes; CAI = 1 indicates perfect adaptation) increasing from pre-optimization values of 0.6–0.8 to near-optimal levels (CAI ≈ 0.95–1.0) post-optimization (Figure 1A,B). Despite this improvement in codon usage, mRNA levels for both codon-optimized sequences (*0432-opt* and *Fluc-opt*) were severely reduced compared to their original sequences regardless of primer positions, as quantified by RT-qPCR using primer sets spanning the entire ORFs (Figure 1C,D).

**Figure 2:**
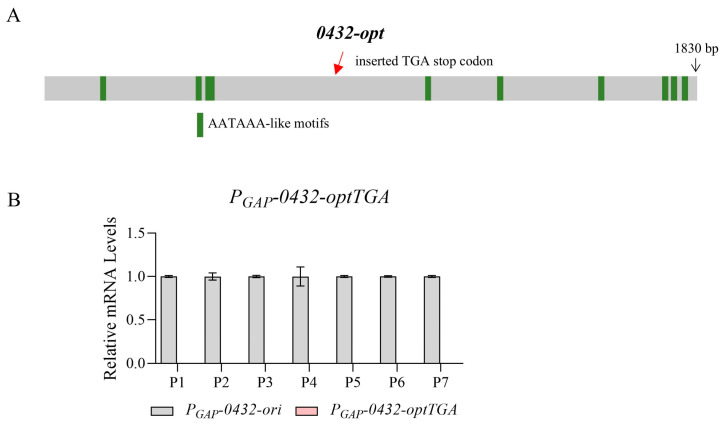
The transcription termination of *0432-opt* is not mediated by AATAAA-like motifs. (**A**) A schematic diagram showing positions of AATAAA-like motifs and the inserted TGA stop codon in *0432-optTGA.* (**B**) RT-qPCR comparing mRNA levels of *0432-ori* and *0432-optTGA*; N = 3. Again, a series of primer sets along the ORF was tested (P1, P2…). For each primer set, the RNA level of the original sequence was set to 1.

As reported, one possible hypothesis is pre-mature transcription termination caused by the accumulation of A/U-rich optimal codons [22]. In eukaryotes, AATAAA-like motifs in coding sequences can trigger aberrant transcriptional termination [40,41]. Predicted by FIMO [42], there are indeed a few sequence motifs along *0432-opt* mimicking the consensus cleavage and polyadenylation signal (Figure 2A; Table S2). However, it cannot explain this well, since the evidence for a shorter mRNA species is not obvious, represented by the relatively low level of mRNAs with 5’ primers (Figure 1A,C, P1 and P2). Another possible scenario is that pre-mature transcription termination happened, and the absence of the stop codon then initiated the decay of the short and abnormal mRNA [43,44]. Since possible premature termination might happen around the position of P3, we then designed the *0432-optTGA* sequence with a stop codon TGA inserted at the end of P3. However, no short mRNA species was identified again (Figure 2B, indicated by the barely visible pink bars). Therefore, additional mechanisms may exist to mediate the poor transcription phenotype of *0432-opt* and *Fluc-opt*.

### 3.2. Codon Optimization Altered Nucleosome Occupancy and Chromatin Accessibility

**Figure 3:**
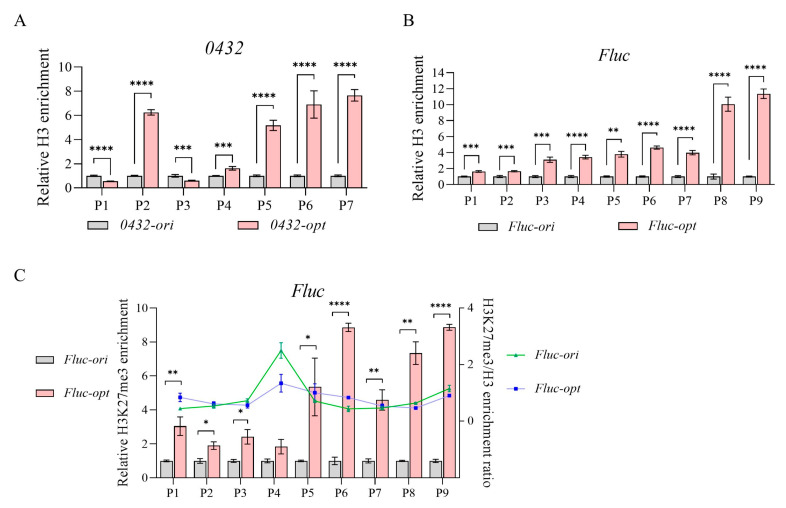
Codon optimization alters nucleosome occupancy. (**A**,**B**) Relative H3 enrichment of *0432* (**A**) and *Fluc* (**B**) before and after codon optimization; N = 3. For each gene, a series of primer sets along the ORF was tested. For each primer set, H3 enrichment of the original sequence was set to 1. (**C**) Relative H3K27me3 enrichment (bar) and H3K27me3/H3 enrichment ratio (line) of *Fluc* before and after codon optimization; N = 3. A series of primer sets along the ORF was tested. For each primer set, H3K27me3 enrichment of the original sequence was set to 1. *p*-values: 0.05–0.01 (*), 0.01–0.001 (**), 0.001–0.0001 (***), and <0.0001 (****).

In order to reveal what caused transcriptional repression in *0432-opt* and *Fluc-opt*, we then examined nucleosome occupancy along their ORFs by histone H3 ChIP. Histone H3, a core component of nucleosomes, serves as a direct marker for nucleosome positioning, as its occupancy reflects the density of nucleosome–DNA interactions. As shown in Figure 3A,B, both *0432-opt* and *Fluc-opt* showed significantly elevated histone H3 occupancy, indicating enhanced nucleosome density across the ORFs. These results provide direct evidence that codon optimization disrupts intrinsic nucleosome-depleted regions, likely through unintended changes in sequence-encoded nucleosome positioning signals.

In addition to nucleosome positioning, the distribution of the histone modification mark H3K27me3, which predominantly is associated with inactive transcription, was also checked. Taking *Fluc* as an example, using ChIP-qPCR normalized to total histone H3 levels, we found no significant differences in H3K27me3/H3 ratios between the original and codon-optimized sequences (Figure 3C). While a weak statistical trend (*p* = 0.032) was observed in the P4 region, the modest magnitude of this change was insufficient to suggest functional consequences for gene expression or chromatin accessibility, consistent with the lack of significant differences across other tested regions. Instead, the observed repression correlates specifically with elevated nucleosome occupancy, suggesting that codon optimization-induced chromatin compaction may act independently of H3K27me3-mediated epigenetic silencing pathways.

**Figure 4:**
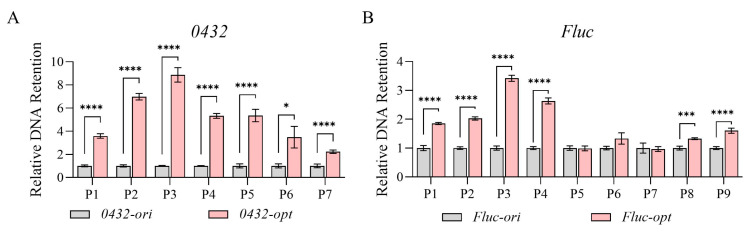
Codon optimization alters chromatin accessibility in *Pichia pastoris*. Relative chromatin DNA retention levels of *0432* (**A**) and *Fluc* (**B**) before and after codon optimization; N = 3. For each gene, a series of primer sets along the ORF was tested. For each primer set, the DNA retention level of the original sequence was set to 1. *p*-values: 0.05–0.01 (*), 0.001–0.0001 (***), and <0.0001 (****).

Since nucleosome positioning was highly correlated with chromatin accessibility, we then performed the DNase I hypersensitivity assay. DNase I preferentially cleaves protein-depleted chromatin regions while leaving protein-bound DNA intact [45]. As shown in Figure 4, *0432-opt* and *Fluc-opt* both showed obviously reduced chromatin accessibility represented by much higher DNA retention ratios. Together, increased nucleosome occupancy and compromised chromatin accessibility may be a contributing factor towards transcriptional repression.

### 3.3. Transcriptional Repression and Reduced Chromatin Accessibility Caused by Codon Optimization Was Not Promoter-Specific

**Figure 5:**
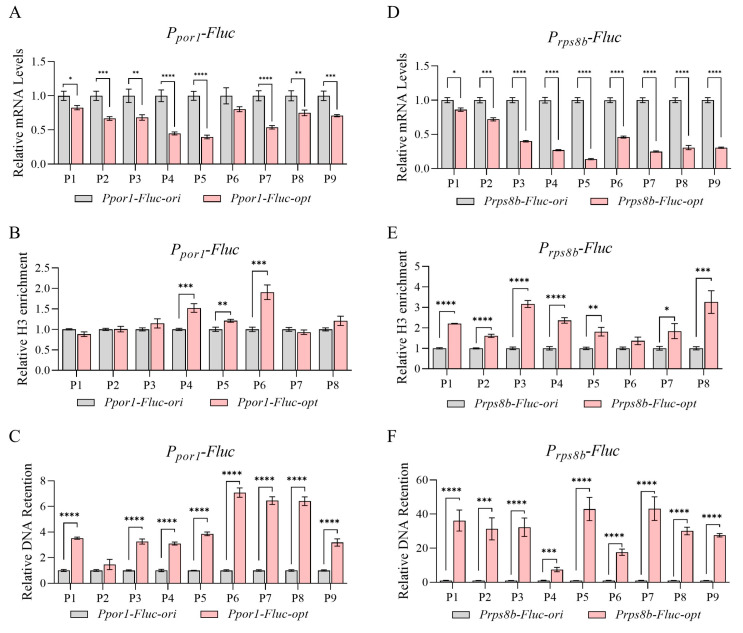
Transcriptional repression and reduced chromatin accessibility caused by codon optimization was not promoter-specific. (**A**–**C**) Relative mRNA levels (**A**), H3 enrichment (**B**), and chromatin DNA retention levels (**C**) of *Fluc-ori/opt* expressed under P*_por1_*; N = 3. (**D**–**F**) Relative mRNA levels (**D**), H3 enrichment (**E**), and chromatin DNA retention levels (**F**) of *Fluc-ori/opt* expressed under P*_rps8b_*; N = 3. *p*-values: 0.05–0.01 (*), 0.01–0.001 (**), 0.001–0.0001 (***), and <0.0001 (****).

Since all the above sequences were expressed under the strong constitutive promoter P*_GAP_*, we then tested their transcription activity under other promoters. Two weaker promoters from *Pichia pastoris* (P*_por1_*, promoter for *PAS_chr2-2_0392*; P*_rpbs8b_*, promoter for *PAS_chr1-1_0439*) were selected here based on previous RNA-seq data [46]. The expression capacity of P*_por1_* was around 64% of P*_GAP_*, while P*_rpbs8b_* was around 30%. After promoter replacement and transgenic strain construction, mRNA levels of *Fluc-ori*/*opt* pairs were checked again. As shown in Figure 5A,D, significant transcription attenuation was also observed when expressed under both promoters. When nucleosome occupancy and chromatin accessibility were further examined, *Fluc-opt* again exhibited elevated nucleosome occupancy (Figure 5B,E) and largely reduced chromatin accessibility (Figure 5C,F). To conclude, altered nucleosome density and chromatin accessibility are likely generated by sequence change mediated by codon optimization, which is not a promoter-driven effect.

## 4. Discussion

As a widely adopted strategy, codon optimization has been extensively documented to improve protein expression in numerous studies. However, the yields vary across genes, with some exhibiting marginal gains or transcriptional problems. Here, we focused on two gene candidates with particularly poor transcription after codon optimization. In addition to the previously reported mechanisms, altered chromatin accessibility was identified as one more effect caused by codon optimization to attenuate transcription.

As revealed by our study, codon replacement affected nucleosome occupancy and chromatin accessibility along ORFs in a promoter-independent manner. This suggests that the ORF sequence itself is correlated with this. It is worth exploring what kind of sequence motifs or codon context is crucial. As predicted by the XSTREME tool (Version 5.5.7) [47], common motifs including “AATTCTTCTTCAAAT” and “AGAAGTTGGAGAAGC” were identified from *0432-opt* and *Fluc-opt* compared to their original sequences. Therefore, further studies are needed to verify the correlation between a specific motif and transcription. If true, these motifs should be circumvented during codon optimization design to prevent transcriptional problems.

Some studies have shown that nucleosome density is negatively correlated with chromatin accessibility [48]. However, this relationship may not be strictly linear, as chromatin accessibility is also influenced by nucleosome stability and dynamic remodeling. For instance, MNase titration experiments revealed that regions with high nucleosome occupancy (e.g., gene bodies) can exhibit moderate-to-high accessibility if nucleosomes are dynamically unstable, whereas low-occupancy heterochromatic regions may remain inaccessible due to tight chromatin compaction [49]. These findings suggest that nucleosome density alone cannot fully predict accessibility; factors such as histone variants (e.g., H2A.Z), post-translational modifications, and chromatin-associated proteins/RNAs synergistically modulate nucleosome turnover and DNA exposure. While our data indicate that H3K27me3 does not play a significant role in mediating the observed expression differences at the tested loci, its functional relevance may vary across distinct genomic contexts or under different cellular conditions. Additionally, other histone modifications (e.g., H3K4me3, H3K9ac) or combinatorial epigenetic marks could contribute to locus-specific accessibility changes. Thus, chromatin accessibility reflects a balance between nucleosome occupancy, stability, and auxiliary regulatory components. In our other studies, we observed that certain genes exhibit strong retention phenotypes after DNase I treatment, but only with a slightly higher level of nucleosome occupancy, indicating additional components and mechanisms may be involved. In addition, chromatin accessibility is only one of the contributing factors to regulate transcription. In fact, the extent of transcription attenuation was slightly different with distinct promoters (Figure 1D and Figure 5A,B). Synonymous codon choice may also act through other possible ways.

In addition to chromatin accessibility, mRNA stability and turnover rates are also critical determinants of transcript abundance. The role of codon optimization on mRNA stability is controversial. While generally expected to stabilize mRNA, exceptions existed for short coding sequences (<500 bp) [50]. Notably, in *Pichia pastoris*, the codon optimization of select genes did not universally increase or reduce stability [19]. We then used the mFold tool to predict the mRNA stabilities of *0432* and *Fluc* before and after codon optimization. As shown in Table S3, the folding energy (represented by ΔG values) was reduced by 10% to 30%, suggesting slightly compromised mRNA stability. Taken together, mRNA stability change may also be a contributing factor towards the gene expression problems of *0432* and *Fluc*. However, it should not be the primary one due to the severe phenotypes on transcription attenuation.

In summary, this work establishes that codon optimization regulates transcription pre-emptively by modulating chromatin accessibility, challenging the conventional view of codon optimization as a purely translation-centric strategy. Our findings provide a mechanistic framework to explain codon optimization failures and open new avenues for refining synthetic gene design.

## Data Availability

The original contributions presented in the study are included in the article, further inquiries can be directed to the corresponding author.

## Supplementary Materials

The following supporting information can be downloaded at: https://www.mdpi.com/article/10.3390/jof11040282/s1, Table S1: Primer sequences used in this study; Table S2: AATAAA-like motifs along *0432-opt*; Table S3: Prediction results of mRNA stability using mFold.
