# Supplementary material for: DNA Sequence Changes Resulting from Codon Optimization Affect Gene Expression in Pichia pastoris by Altering Chromatin Accessibility
Source: J Fungi (Basel). 2025 Apr 3;11(4):282. doi: 10.3390/jof11040282 (PMC12029099; doi:10.3390/jof11040282)
Supplement: Supplementary file 1 [file jof-11-00282-s001.zip › jof-3556782-supplementary.pdf]

Table S1 Primer sequences used in this study

primers for *0432*		
	ori	opt
P1	F: AATTCGACGGTAAAAGGAAGGCCCAAA	F: AATTCTACTGTTAAAGGAAGACCAAAA
	R: TTAGCACTAGAGGACAGTTCTGGAA	R: TTAGCAGAAGAAGACAATTCTGGAA
P2	F: TTCCAGAACTGTCCTCTAGTGCTAA	F: TTCCAGAATTGTCTTCTTCTGCTAA
	R: GTCCAGGGTAGTCAAATCAAATTC	R: ATCCAAAGTAGTCAAATCAAATTC
P3	F: CAGAAGAGCAAGATCAAAGTCAGTG	F: CTGAAGAACAAGATCAATCTCAATG
	R: AAAGGTTGTATTCCAACAGCCC	R: AATGGTTGAATTCCAACAGCTC
P4	F: GGGCTGTTGGAATACAACCTTT	F: GAGCTGTTGGAATTCAACCATT
	R: CGTTGGAATGCTATTTCTTGGATT	R: AGTTGGAATAGAATTTCTTGGATT
P5	F: AATCCAAGAAATAGCATTCCAACG	F: AATCCAAGAAATTCTATTCCAACTTC
	R: GGCCTGGCATCAATCATACGCAT	R: GGTCTAGCATCAATCATTCTCAT
P6	F: ATGCGTATGATTGATGCCAGGCC	F: ATGAGAATGATTGATGCTAGACC
	R: CCCCATGGTTGGAGGTGATAT	R: TCCCATAGTTGGTGGAGAAAT
P7	F: ATATCACCTCCAACCATGGGG	F: ATTTCTCCACCAACTATGGGA
	R: AGAAGCCTCATCAGATATCGCA	R: AGAAGCTTCATCAGAAATAGCA
primers for *Fluc*		
	ori	opt
P1	F: GGAACAATTGCTTTTACAGATGCACATAT	F: GGAACTATTGCTTTTACTGATGCTCATAT
	R: GGCGCAACTGCAACTCCGATAAA	R: GGAGCAACAGCAACTCCAATAAA
P2	F: TTTATCGGAGTTGCAGTTGCGCC	F: TTTATTGGAGTTGCTGTTGCTCC
	R: GAAATCCCTGGTAATCCGTTTTAGAATCC	R: GAAATCCTTGATAATCAGTTTTAGAATCC
P3	F: GGATTCTAAAACGGATTACCAGGGATTTC	F: GGATTCTAAAACTGATTATCAAGGATTTC
	R: CAGGCAGTTCTATGAGGCAGAGC	R: CAAGCAGTTCTATGTGGCAAAGC

P4	F: GCTCTGCCTCATAGAACTGCCTG	F: GCTTTGCCACATAGAACTGCTTG
	R: CTCAGAAACAGCTCTTCTTCAAATCTATACAT	R: CTCAAAAACAATTCTTCTTCAAATCTATACAT
P5	F: ATGTATAGATTTGAAGAAGAGCTGTTTCTGAG	F: ATGTATAGATTTGAAGAAGAATTGTTTTTGAG
	R: GCAACCGCTTCCCCGACTTC	R: GCAACAGCTTCTCCAACTTC
P6	F: GAAGTCGGGGAAGCGGTTGC	F: GAAGTTGGAGAAGCTGTTGC
	R: TTCGCCTCTTTGATTAACGCCCA	R: TTCTCCTCTTTGATTAACTCCCA
P7	F: TGGGCGTTAATCAAAGAGGCGAA	F: TGGGAGTTAATCAAAGAGGAGAA
	R: GATTCCAATTCAGCGGGAGCCAC	R: GATTCCAATTCAGCTGGAGCAACTT
P8	F: GTGGCTCCCGCTGAATTGGAATC	F: GTTGCTCCAGCTGAATTGGAATC
	R: ACAAACACAACTCCTCCGCGCAA	R: ACAAAAACAACTCCTCCTCTCAA
P9	F: TTGCGCGGAGGAGTTGTGTTTGT	F: TTGAGAGGAGGAGTTGTTTTTGT
	R: GCGATCTTTCCGCCCTTCTT	R: CAGCAATTTTTCCTCCTTTTTTAGC

Table S2 AATAAA-like motifs along *0432-op*t

Motif ID	Alt ID	Sequence Name	Strand	Start	End	p-value	q-value	Matched Sequence
1	AATAAA	0432-opt	+	157	162	0.000475	0.504	AATAAA
1	AATAAA	0432-opt	+	373	378	0.00653	0.504	AATGAA
1	AATAAA	0432-opt	+	424	429	0.00653	0.504	GATAAA
1	AATAAA	0432-opt	+	454	459	0.00653	0.504	TATAAA
1	AATAAA	0432-opt	+	1054	1059	0.00653	0.504	ACTAAA
1	AATAAA	0432-opt	+	1270	1275	0.00653	0.504	AATAGA
1	AATAAA	0432-opt	+	1534	1539	0.00653	0.504	AATAGA
1	AATAAA	0432-opt	+	1724	1729	0.00653	0.504	AATATA
1	AATAAA	0432-opt	+	1750	1755	0.00653	0.504	ACTAAA

1	AATAAA	0432-opt	+	1774	1779	0.00653	0.504	ATTAAA

Table S3 Prediction results of mRNA stability using mFold

Parameter	0432-ori	0432-opt	Fluc-ori	Fluc-opt
ΔG (kcal/mol)	-527.80	-466.20	-516.40	-387.20
Base Pairing Rate	3.7%	5.1%	6.1%	2.3%
ss-count Value	18.23±17.13	16.17±14.89	16.09±13.85	18.55±15.19
A/U Content	1037 (538A+499U)	1264 (559A+705U)	892 (459A+433U)	1138 (518A+620U)
G/C Content	811 (426C+385G)	584 (257C+327G)	779 (410G+369C)	533 (339G+194C)

Gene sequences used in this study:

0432-ori

ATGTCCAAAACAAATTCGACGGTAAAAGGAAGGCCCAAACTAGTTCAATCTGTGAACGGATTTTTTCCAAAGGTGCGAATGTGGTTACACACGGACGATTTTTT
CATCCGAACCTTCTAAAAGTGAAGATTCAGGTCTGGATTTTGTTAAACCCAATAAATGGTTCAACTTACTGGTTCCAGAACTGTCCTCTAGTGCTAAGATTGCATTA
TCTCGTTGGAAAGGTAATGACCTACTAACGATCCCACCCATGGTTTTAGAAGTTTTTCTGGTAGTTCCAACAGAGCATTCAGATAGTCTGGTCCTTCGTGGGGGA
GCCGATGGATTGAGCCAAGATGATGTCGTCATCAACGTTGGTAACTACGAGCGCAACGAGATTGTTCTAGAACGATGGTTACTAGAATTTGATTTGACTACCCTG
GACAAAAATTCTACTGAGATCTACGGTATTTACAAGAAAATGATCATTCTTTTTCGCAATCTCTACACATTTGTACGATTGATGCCTGCATTTAGGCTCTTTCAAGAG
GGGAACTGGAAAATAGGGACTCGAACATTAGATGGTAGCGAGCCAATCTCCAGCAAAGATCGAATTGGTTTAAGTGATTCGTTCCTGGGCGAATCGAAGAATTCA
GAAGAGCAAGATCAAAGTCAGTGCTATTATTCCCATTTGAGCCAAAAAAAATTTCGATCTATAACCACTTCAATGGGTAGTTTGAAGATCAGTTGCAGCTTCCGTAG
AAATACTGCCTTTCGGTGGGCAAGCACACGTCGAAAAGCGCGTGATAACACGGTCACAGGTGGGCTTTCTCGGGCTGTTGGAATACAACCTTTCAAGACTGGT
GTTTTATCGTCCAGCCCTGGTCGTAGTCCCGGTCATGGCTTCACTGCGACACTTTGTGCCCGGACTGAGTCACAGCCTATCCCTCTTCAAATTCATCATCGCTCA
AGCTCTAACGCTTCCCTTGTACAACTTCTTCGGAATCCAAGAAATAGCATTCCAACGTCAATAAACTCCGTGCTTGAGGACCATTCAACAATCTCTCCAGCAACAA
AATTCTCTTCCTCTTTTAGGTTAGCACGCAGAGGGAGTCTCCATTCCCGAACATCGGTCGATCGTCGTTCAAGTGACTTATCATCGTCTGACATAGACCCAGACC
AATTCTACGTCGATGAAGATATAAATGACCTCATGCGTATGATTGATGCCAGGCCGAATTTAAGGCTTTCCTCAGCACGGTCACGTGAAACTTCACCGTCATCTCT
GAATCGATTTCAACTCTTACAGAAAACTCATGATATCTTGAGTGATTCGGTACATGCAAGTTTACCCACAAGTGCCCATGGAGTTCTCTCATTGTCCCCATCACGA

AGGTATTCCATATCACCTCCAACCATGGGGCCAGGCTCATCTACAGCCTCAATCTCTCAATCTTTAACTTACGCACGAATGGATCACCAGGACTCCGCACAATCA GCCAATTCTATCCGTGACATTTTGCAGAGTTCGAGCCGACGTAACTCTTCTTCCAACAGACGAGGATCTGGCCAGTCGCCAAGGCCTGGAACAATACTAGGACT ACCATCCGGGCTAGGCTCTGGTGACAGTGCGATATCTGATGAGGCTTCTAAAGAACATGTTTATGAAGAGCACGCAATAATTGATGATGATGAGGAGGAGGAAG ACATGATCACTCAAAGAAATTTGAATCATCTATTGAAGTACAGAAAACTTCGCCTTCAAGATACGAAAAAAAGCAAAGAGATCACGATCAAAGAAGAGGATGACGA TTTGTTGTTCACAATGAGCGACATGAATCTTAGCCATCATCATCATCATCATTGA

0432-opt

ATGTCTAAAACTAATTCTACTGTTAAAGGAAGACCAAAATTGGTTCAATCTGTTAATGGATTTTTTTCTAAAGGAGCTAATGTTGTTACTCATGGAAGATTTTTTTCTT CTGAACCATCTAAATCTGAAGATTCTGGATTGGATTTTGTTAAACCAAATAAATGGTTTAATTTGTTGGTTCCAGAATTGTCTTCTTCTGCTAAAATTGCTTTGTCTAG ATGGAAAGGAAATGATTTGTTGACTATTCCACCAATGGTTTTGGAAGTTTTTTTGGTTGTTCCAACTGAACATTCTGATTCTTTGGTTTTGAGAGGAGGAGCTGAT GGATTGTCTCAAGATGATGTTGTTATTAATGTTGGAAATTATGAAAGAAATGAAATTGTTTTGGAAAGATGGTTGTTGGAATTTGATTTGACTACTTTGGATAAAAATT CTACTGAAATTTATGGAATTTATAAAAAAATGATTATTTTGTTTAGAAATTTGTATACTTTTGTTAGATTGATGCCAGCTTTTAGATTGTTTCAAGAAGGAAATTGGAAA ATTGGAACTAGAACTTTGGATGGATCTGAACCAATTTCTTCTAAAGATAGAATTGGATTGTCTGATTCTTTTTTGGGAGAATCTAAAAATTCTGAAGAACAAGATCAA TCTCAATGTTATTATTCTCATTTGTCTCAAAAAAAAATTTAGATCTATTACTACTTCTATGGGATCTTTGAAAATTTCTTGTTCTTTTAGAAGAAATACTGCTTTTAGATGG GCTTCTACTAGAAGAAAAGCTAGAGATAATACTGTTACTGGAGGATTGTCTAGAGCTGTTGGAATTCAACCATTTAAAACTGGAGTTTTGTCTTCTTCTCCAGGAAG ATCTCCAGGACATGGATTTACTGCTACTTTGTGTGCTAGAACTGAATCTCAACCAATTCCATTGCAAATTCATCATAGATCTTCTTCTAATGCTTCTTTGGTTCAATT GTTGAGAAATCCAAGAAATTCTATTCCAACTTCTATTAATTCTGTTTTGGAAGATCATTCTACTATTTCTCCAGCTACTAAATTTTCTTCTTCTTTTAGATTGGCTAGAA GAGGATCTTTGCATTCTAGAACTTCTGTTGATAGAAGATCTTCTGATTTGTCTTCTTCTGATATTGATCCAGATCAATTTTATGTTGATGAAGATATTAATGATTTGAT GAGAATGATTGATGCTAGACCAAATTTGAGATTGTCTTCTGCTAGATCTAGAGAAACTTCTCCATCTTCTTTGAATAGATTTCAATTGTTGCAAAAAACTCATGATAT TTTGTCTGATTCTGTTCATGCTTCTTTGCCAACTTCTGCTCATGGAGTTTTGTCTTTGTCTCCATCTAGAAGATATTCTATTTCTCCACCAACTATGGGACCAGGATC TTCTACTGCTTCTATTTCTCAATCTTTGACTTATGCTAGAATGGATCATCAAGATTCTGCTCAATCTGCTAATTCTATTAGAGATATTTTGCAATCTTCTTCTAGAAGAA ATTCTTCTTCTAATAGAAGAGGATCTGGACAATCTCCAAGACCAGGAACTATTTTGGGATTGCCATCTGGATTGGGATCTGGAGATTCTGCTATTTCTGATGAAGC TTCTAAAGAACATGTTTATGAAGAACATGCTATTATTGATGATGATGAAGAAGAAGAAGATATGATTACTCAAAGAAATTTGAATCATTTGTTGAAATATAGAAAATTG AGATTGCAAGATACTAAAAAAATCTAAAGAAATTACTATTAAAGAAGAAGATGATGATTTGTTGTTTACTATGTCTGATATGAATTTGTCTCATCATCATCATCATCATTG A

0432-optTGA

ATGTCTAAAACTAATTCTACTGTTAAAGGAAGACCAAAATTGGTTCAATCTGTTAATGGATTTTTTTCTAAAGGAGCTAATGTTGTTACTCATGGAAGATTTTTTTCTT

CTGAACCATCTAAATCTGAAGATTCTGGATTGGATTTTGTTAAACCAAATAAATGGTTTAATTTGTTGGTTCCAGAATTGTCTTCTTCTGCTAAAATTGCTTTGTCTAG
ATGGAAAGGAAATGATTTGTTGACTATTCCACCAATGGTTTTGGAAGTTTTTTTGGTTGTTCCAACTGAACATTCTGATTCTTTGGTTTTGAGAGGAGGAGCTGAT
GGATTGTCTCAAGATGATGTTGTTATTAATGTTGGAAATTATGAAAGAAATGAAATTGTTTTGGAAAGATGGTTGTTGGAATTTGATTTGACTACTTTGGATAAAAATT
CTACTGAAATTTATGGAATTTATAAAAAAATGATTATTTTGTTTAGAAATTTGTATACTTTTGTTAGATTGATGCCAGCTTTTAGATTGTTTCAAGAAGGTAATTGGAAA
ATTGGAACTAGAACTTTGGATGGATCTGAACCAATTTCTTCTAAAGATAGAATTGGATTGTCTGATTCTTTTTTGGGAGAATCTAAAAATTCTGAAGAACAAGATCAA
TCTCAATGTTATTATTCTCATTTGTCTCAAAAAAAATTTAGATCTATTACTACTTCTATGGGATCTTTGAAAATTTCTTGTTCTTTTAGAAGAAATACTGCTTTTAGATGG
GCTTCTACTAGAAGAAAAGCTAGAGATAATACTGTTACTGGAGGATTGTCTTGAAGAGCTGTTGGAATTCAACCATTTAAAACTGGAGTTTTGTCATCTTCTCCAGG
AAGATCTCCAGGACATGGACTTACTGCTACTTTGTGTGCTAGAACTGAATCTCAACCAATTCCATTGCAAATTCATCATAGATCTTCTTCTAATGCTTCTTTGGTTCA
ATTGTTGGGAAATCCAAGAAATTCTATTCCAACTTCTATTAATTCTGTTTTGGAAGATCATTCTACTATTTCTCCAGCTACTAAATTTTCTTCTTCTTTTAGATTGGCTA
GAAGAGGATCTTTGCATTCTAGAACTTCTGTTGATAGAAGATCTTCTGATTTGTCTTCTTCTGATATTGATCCAGATCAATTTTATGTTGATGAAGATATTAATGATTT
GATGAGAATGATTGATGCTAGACCAAATTTGAGATTGTCTTCTGCTAGATCTAGAGAAACTTCTCCATCTTCTTTGAATGGATTTCAATTGTTGCAAAAAACTCATGA
TATTTTGTCTGATTCTGTTCATGCTTCTTTGCCAACTTCTGCTCATGGAGTTTTGTCTTTGTCTCCATCTAGAAGATATTCTATTTCTCCACCAACTATGGGACCAGG
ATCTTCTACTGCTTCTATTTCTCAATCTTTGACTTATGCTAGAATGGATCATCAAGATTCTGCTCAATCTGCTAATTCTATTAGAGATATTTTGCAATCTTCTTCTAGAA
GAAATTCTTCTTCTAATAGAAGAGGATCTGGACAATCTCCAAGACCAGGAACTATTTTGGGATTGCCATCTGGATTGGGATCTGGAGATTCTGCTATTTCTGATGA
AGCTTCTAAAGAACATGTTTATGAAGAACATGCTATTATTGATGATGATGAAGAAGAAGAAGATATGATTACTCAAAGAAATTTGAATCATTTGTTGAAATATAGAAAA
TTGAGATTGCAAGATACTAAAAAATCTAAAGAAATTACTATTAAAGAAGAAGATGATGATTTGTTGTTTACTATGTCTGATATGAATTTGTCTCATCATCATCATCATCA
TTGA
Fluc-ori
ATGGAAGACGCCAAAAACATAAAGAAAGGCCCGGCGCCATTCTATCCGCTGGAAGATGGAACCGCTGGAGAGCAACTGCATAAGGCTATGAAGAGATACGCCC
TGGTTCCTGGAACAATTGCTTTTACAGATGCACATATCGAGGTGGACATCACTTACGCTGAGTACTTCGAAATGTCCGTTCGGTTGGCAGAAGCTATGAAACGATA
TGGGCTGAATACAAATCACAGAATCGTCGTATGCAGTGAAAACTCTCTTCAATTCTTTATGCCGGTGTTGGGCGCGTTATTTATCGGAGTTGCAGTTGCGCCCGC
GAACGACATTTATAATGAACGTGAATTGCTCAACAGTATGGGCATTTCGCAGCCTACCGTGGTGTTCGTTTCCAAAAAGGGGTTGCAAAAAATTTTGAACGTGCAA
AAAAAGCTCCCAATCATCCAAAAAATTATTATCATGGATTCTAAAACGGATTACCAGGGATTTCAGTCGATGTACACGTTCGTCACATCTCATCTACCTCCCGGTTT
TAATGAATACGATTTTGTGCCAGAGTCCTTCGATAGGGACAAGACAATTGCACTGATCATGAACTCCTCTGGATCTACTGGTCTGCCTAAAGGTGTCGCTCTGCCT
CATAGAACTGCCTGCGTGAGATTCTCGCATGCCAGAGATCCTATTTTTGGCAATCAAATCATTCCGGATACTGCGATTTTAAGTGTTGTTCCATTCCATCACGGTTT
TGGAATGTTTACTACACTCGGATATTTGATATGTGGATTTCGAGTCGTCTTAATGTATAGATTTGAAGAAGAGCTGTTTCTGAGGAGCCTTCAGGATTACAAGATTC

AAAGTGCGCTGCTGGTGCCAACCCTATTCTCCTTCTTCGCCAAAAGCACTCTGATTGACAAATACGATTTATCTAATTTACACGAAATTGCTTCTGGTGGCGCTCC
CCTCTCTAAGGAAGTCGGGGAAGCGGTTGCCAAGAGGTTCCATCTGCCAGGTATCAGGCAAGGATATGGGCTCACTGAGACTACATCAGCTATTCTGATTACAC
CCGAGGGGGATGATAAACCGGGCGCGGTCGGTAAAGTTGTTCCATTTTTTGAAGCGAAGGTTGTGGATCTGGATACCGGGAAAACGCTGGGCGTTAATCAAAG
AGGCGAACTGTGTGTGAGAGGTCCTATGATTATGTCCGGTTATGTAAACAATCCGGAAGCGACCAACGCCTTGATTGACAAGGATGGATGGCTACATTCTGGAG
ACATAGCTTACTGGGACGAAGACGAACACTTCTTCATCGTTGACCGCCTGAAGTCTCTGATTAAGTACAAAGGCTATCAGGTGGCTCCCGCTGAATTGGAATCCA
TCTTGCTCCAACACCCCAACATCTTCGACGCAGGTGTCGCAGGTCTTCCCGACGATGACGCCGGTGAACTTCCCGCCGCCGTTGTTGTTTTGGAGCACGGAAA
GACGATGACGGAAAAAGAGATCGTGGATTACGTCGCCAGTCAAGTAACAACCGCGAAAAAGTTGCGCGGAGGAGTTGTGTTTGTGGACGAAGTACCGAAAGGT
CTTACCGGAAAACTCGACGCAAGAAAAATCAGAGAGATCCTCATAAAGGCCAAGAAGGGCGGAAAGATCGCCGTTCATCATCATCATCATCATTGA

Fluc-opt ：

ATGGAAGATGCTAAAAATATTAAAAAAGGACCAGCTCCATTTTATCCATTGGAAGATGGAACTGCTGGAGAACAATTGCATAAAGCTATGAAAAGATATGCTTTGGT
TCCAGGAACTATTGCTTTTACTGATGCTCATATTGAAGTTGATATTACTTATGCTGAATATTTTGAAATGTCTGTTAGATTGGCTGAAGCTATGAAAAGATATGGATTG
AATACTAATCATAGAATTGTTGTTTGTTCTGAAAATTCTTTGCAATTTTTTATGCCAGTTTTGGGAGCTTTGTTTATTGGAGTTGCTGTTGCTCCAGCTAATGATATTT
ATAATGAAAGAGAATTGTTGAATTCTATGGGAATTTCTCAACCAACTGTTGTTTTTGTTTCTAAAAAAGGATTGCAAAAAATTTTGAATGTTCAAAAAAAAATTGCCAAT
TATTCAAAAAATTATTATTATGGATTCTAAAACTGATTATCAAGGATTTCAATCTATGTATACTTTTGTTACTTCTCATTTGCCACCAGGATTTAATGAATATGATTTTGTT
CCAGAATCTTTTGATAGAGATAAAACTATTGCTTTGATTATGAATTCTTCTGGATCTACTGGATTGCCAAAAGGAGTTGCTTTGCCACATAGAACTGCTTGTGTTAGA
TTTTCTCATGCTAGAGATCCAATTTTTGGAAATCAAATTATTCCAGATACTGCTATTTTGTCTGTTGTTCCATTTCATCATGGATTTGGAATGTTTACTACTTTGGGAT
ATTTGATTTGTGGATTTAGAGTTGTTTTGATGTATAGATTTGAAGAAGAATTGTTTTTGAGATCTTTGCAAGATTATAAAATTCAATCTGCTTTGTTGGTTCCAACTTT
GTTTTCTTTTTTTGCTAAATCTACTTTGATTGATAAATATGATTTGTCTAATTTGCATGAAATTGCTTCTGGAGGAGCTCCATTGTCTAAAGAAGTTGGAGAAGCTGTT
GCTAAAAGATTTCATTTGCCAGGAATTAGACAAGGATATGGATTGACTGAAACTACTTCTGCTATTTTGATTACTCCAGAAGGAGATGATAAACCAGGAGCTGTTGG
AAAAGTTGTTCCATTTTTTGAAGCTAAAGTTGTTGATTTGGATACTGGAAAAACTTTGGGAGTTAATCAAAGAGGAGAATTGTGTGTTAGAGGACCAATGATTATGT
CTGGATATGTTAATAATCCAGAAGCTACTAATGCTTTGATTGATAAAGATGGATGGTTGCATTCTGGAGATATTGCTTATTGGGATGAAGATGAACATTTTTTTATTGT
TGATAGATTGAAATCTTTGATTAAATATAAAGGATATCAAGTTGCTCCAGCTGAATTGGAATCTATTTTGTTGCAACATCCAAATATTTTTGATGCTGGAGTTGCTGG
ATTGCCAGATGATGATGCTGGAGAATTGCCAGCTGCTGTTGTTGTTTTGGAACATGGAAAAACTATGACTGAAAAAGAAATTGTTGATTATGTTGCTTCTCAAGTTA
CTACTGCTAAAAAATTGAGAGGAGGAGTTGTTTTTGTTGATGAAGTTCCAAAAGGATTGACTGGAAAATTGGATGCTAGAAAAATTAGAGAAATTTTGATTAAAGCT
AAAAAAGGAGGAAAAATTGCTGTTCATCATCATCATCATCATTGA

P_{por1} ：

TTATGCCATCGTGAACACAAATATCACAGGTGCATTTGGTGCTATTTCCTGGTGTCTATTAGACTGGCGTTTGGAGCGCCGTTTCAGTACTGTTGCTCTATGCTCC
GGTGCCATTTCGGGCCTCGTGGCAGCAACTCCAGCCTCAGGTATCATTCCTCTTTGGGCCAGTGTTATTCTTGGTATTGTATCAGGAGTGGTTTGTAACTACGCA
ACCAAGATTAAAGTCATTTGTCGAGTCGATGATTCCATGGATGTTCTAGCAGAGCACGGTATCGCTGGTGTTATTGGTCTCGTCTTCAACGCATTATTTGGGTCGG
CTACTGTCATTGGTTATGATGGCCTTACCGAGCACGAAGGTGGTTGGATAGACCACAACTGGAAACAGTTGTACAAACAGATTGCATTCATTTTTGCTTGTATTGG
ATACTCGATGGCCATCACCGCTCTTATCTGTTTCATCCTCAACCGTATTCCATTTTTGCAACTGCGAGCTTCAGAAGAGGCTGAGGAGAAAGGTATGGATGAGGA
TCAGATTGGAGAGTTCGCTTATGACTACGTGGAAGTACGTCGTGATTTTTTGGCTTGGGGATCAGGCCCAAACAATGGCTTCAAGGAGCCGGAAGTTCTGGATC
AGGTAGTTCCGGTTAATGATTTCAGCAGTGACCAGAATGTGACTAATGAGACCAACGAATCTGAGAAGCAGTAGAGTAAATATAGAGATGATATTTAGTGTATTCTA
ATGCTTATGTAATGTATTAAGCAAAAAGTTGTGTTTATGAGTTAGCATTTGTCTTAGCAAACATAAAATTATGTCGACATTTGCAACCCGCATGTCTAGTGTTTTTAGA
TCGATCTTCGATGTGTAGAATAATAGCCTCCACGTGATGCCCCGCGATTTTGTTGGGTCTCAATGCCTCCAACATAAACCCATCACGTATAAAAAGCCCTCTTAAC
CCTCCCCCCTGTTTCGTTTGCTTCATCACTTAACCTGAACTATCAAA

P_{rpbs8b}

CTGTCCCACTGATACTGCTGAAGGTGCTATCAATATTGGAGAAATAGAAGATCCAGAAGATAAAGTGCGATACCAAATATGGAAAGAAAATGAATCCAAACGAGCA
GACCAGAACGATAAATCGCCTGCATCAATCCTTTCAAAATTGCAGTACATATTGTTTTTTATATTTCTGGGTGCCATATTCAAGTATTATGTTCATTGATTGTTTTAAAA
TGTGACCTTTAGTCAAACTTTCATGAAAGAAAAGCGGCTAACTGGCTAAAGTTTATGGCCTATTTCAGAAAGCTAGAACCCAAAACTGGTTTTTCTCCTCTGATTGC
TAAACTCTTGAGTACATCCCGATCGTAGTGTTGATAGTCGTTGCTTTGTTGACTTCGTCTCAGCCCTTTTGTGAGCTGTCGTCCATAATCTCAGTGATTCATTTCAT
AAGCATATTTTAAATGTAATATGTTAACTTAGTTGATCGACATATTGTTGCAAATCTTCTTCTTCAAAAACTACTTGAACCATTAGAGAGCAAGATGGACAAATAGCAA
CATCTTCCCCGTCCTTTAAATCATCTAAGGAGATTTGAAACCTATCCCCACAAGGACATGGATACTGGAAAATCTGTAGAATAGGATCAAAAATAAAATCTTCGATTT
CTATATGATCATAAATTGTTTCCATGAATAGTATTAAAACCTTTGAGTACACCTATATGTCACTGGATGCTTCTTACAGATTCCTCATGTAGAACCGATAAAAAGTAAA
GGATCATACAAAAAGATAGAAAAATAACAATCACGTGAGTAGATAAAGGGTAAAATAATCTTAAAAGTCACAACATCTTCAATCGGTAATTAACTTGGTCATGTGATT
TGGCTCACTTGACTTCGCTTCTAAAAATCTTTTCATACTTCTCTTTCCTTAAACATCAAGTAAAGTAAGTAAATTACCAGAACTTGCGAAATCAAGTCATTGCTCCATT
CAACTTCATCGGCTAACAGTTATTAGA
